# *In vitro* screening and evaluation of antivenom phytochemicals from *Azima tetracantha* Lam. leaves against *Bungarus caeruleus* and *Vipera russelli*

**DOI:** 10.1186/1678-9199-20-12

**Published:** 2014-04-01

**Authors:** Bhavya Janardhan, Vineetha M Shrikanth, Kiran K Mirajkar, Sunil S More

**Affiliations:** 1 Department of Biochemistry, Centre for Post Graduate Studies, Jain University, Jayanagar 3rd block, Bangalore, Karnataka 560011, India; 2 Department of Biochemistry, University of Agricultural Sciences, Dharwad 580007, India

**Keywords:** Acetylcholinesterase, *Azima tetracantha*, Antivenom, Krait, Viper, *In vitro*

## Abstract

**Background:**

Snakebites are considered a neglected tropical disease that affects thousands of people worldwide. Although antivenom is the only treatment available, it is associated with several side effects. As an alternative, plants have been extensively studied in order to obtain an alternative treatment. In folk medicine, *Azima tetracantha* Lam. is usually used to treat snakebites. The present study aims to provide a scientific explanation for the use of this plant against snakebite. The extracts of shade dried leaves of *A. tetracantha* were tested for *in vitro* inhibitory activity on toxic venom enzymes like phosphomonoesterase, phosphodiesterase, acetylcholinesterase, hyaluronidase etc. from *Bungarus caeruleus* and *Vipera russelli* venoms.

**Results:**

The ethylacetate extract rendered a significant inhibitory effect on the phosphomonoesterase, phosphodiesterase, phospholipase A_2_ and acetylcholinesterase enzymes.

**Conclusions:**

The present study suggests that ethylacetate extract of *A. tetracantha* leaves possesses compounds that inhibit the activity of toxic enzymes from *Bungarus caeruleus* and *Vipera russelli* venom. Further pharmacological and *in vivo* studies would provide evidence that this substance may lead to a potential treatment against these venoms.

## Background

Snakebite remains a major health concern throughout the world especially in India, due to the high mortality rate in the country. Worldwide, snake envenomation incidence exceeds 5 million affected people per year [1]. In Asia, an estimated four million snakebites occur every year, of which approximately 50% of the victims are envenomed, resulting in 100,000 annual deaths [2]. In India alone about 35,000 to 50,000 deaths occur annually. The major families of snakes in India are Elapidae, Viperidae and Hydrophidae. The most common poisonous snakes in the country are cobra (*Naja naja*), krait (*Bungarus caeruleus*), Russell’s viper (*Daboia russelli*) and saw-scaled viper (*Echis carinatus*). The former two belong to Elapidae and the latter two belong to Viperidae [3]. The venoms of cobra and krait are neurotoxic, that is, they affect the victim’s central nervous system and cause heart failure. Their venom possesses several proteins, including cardiotoxins, neurotoxins and phospholipase A_2_, that are responsible for their toxicity. The venoms of Russell’s viper and saw-scaled viper are histotoxic and hemorrhagic, therefore they provoke hemorrhagic manifestations that include epistaxis and cardiac manifestations such as myocarditis and cardiac failure [4].

Presently, antivenom immunotherapy is the only treatment available against snake envenomation. The side effects of antivenom include anaphylactic shock, pyrogen reaction and serum sickness. These symptoms are possibly outcomes of the action of non-immunoglobulin proteins present in higher concentrations in antivenom [5]. In this perspective, several attempts have been made to develop snake venom antagonists from plants. In folk medicine, plant drug recipes are passed on to generations by oral tradition and are used as antidotes [6]. Considering the limitations of antivenom, investigations on plants are based on the fact that some of their extracts are rich in bioactive compounds with promising antivenom effects [7,8].

*Hemidesmus indicus* root extract and methanolic leaf extract of *Azadirachta indica* have been proved to neutralize phospholipase A_2_ activity induced by Russell’s viper venom [9,10]. *Mimosa pudica* has shown antihyaluronidase activity against *Naja naja*, *Vipera russelli* and *Echis carinatus* venoms [11]. *Echis carinatus* enzymatic effects have been inhibited by the extracts of *Andrographis paniculata* and *Aristolochia indica*. Enzymatic and pharmacological activities of phospholipase A_2_ induced by *Vipera russelli* venom have been inhibited by aristolochic acid from *Aristolochia radix*[12,13].

*In vitro* tests with polyphenols from *Areca catechu* L. and *Quercus infectoria* Oliv. showed inhibition of phospholipase A_2_, proteases, hyaluronidase and L-amino acid oxidase of *Naja naja kaouthia* and *Calloselasma rhodostoma* venoms [14]. In addition, *Tamarindus indica* has shown potent venom neutralizing properties [15].

*Azima tetracantha* Lam*.* belongs to the Salvodoraceae family, it is known as Kundali in Ayurvedic medicine and also called uppimullu in kannada [16,17]. Antidiarrheal activity has been reported in this plant and antimicrobial activity in its fruits [18,19]. Moreover, there are reports that the juice of its leaves is efficient against toothache and earache [20]. In Indian tribes, leaf paste of *A. tetracantha* is used to treat snakebites [17]. The presence of dimeric piperdine alkaloids azimine, azacarpaine, carpaine, triterpenoids, isorhamnetin 3-rutinoside, neoascorbinogen and glucosinolates and novel fatty acids have been reported as some of the phytochemicals present in the plant [21-25]. The present study investigates the neutralizing activity of *A. tetracantha* plant extracts on krait and Russell’s viper venom toxic enzymes by *in vitro* methods.

## Methods

### Venom

The lyophilized venoms of *Bungarus caeruleus* and *Vipera russelli* were obtained from Irula Snake Catcher’s Cooperative Society, Kancheepuram, Chennai. Snake venom (5 mg/mL) was dissolved in physiological saline and centrifuged at 2000 g for ten minutes. The supernatant was used for further analysis and stored at 4°C. The protein concentration was estimated according to the method of Lowry *et al*. [26].

5′ Adenosine mono phosphate (5′ AMP), disodium-p-nitrophenol phosphate, L-leucine, diansidine hydrochloride, horseradish peroxidase, 5,5′-dithiobis-(2-nitrobenzoic acid) (DTNB), acetylthiocholine iodide, hyaluronic acid, cetyltrimethylammonium bromide, lecithin were purchased from Himedia Laboratories (India) and casein from Sigma Aldrich Laboratories (USA). All the other reagents were of analytical grade.

### Plants and extraction

Fresh leaves of *A. tetracantha* were collected in September in Kurubarahatty village, Chitradurga district, Karnataka, India. The plant specimens were identified and authenticated by the National Ayurveda Dietetics Research Institute, Bangalore, Karnataka (Drug Authentication/SMPU/NADRI/BNG/2013-2014/765). The leaves were thoroughly washed in order to remove adhering dust, shade dried and ground into powder for further use.

Powdered material was prepared in soxhlet apparatus using petroleum ether (60-80°C), hexane, chloroform, ethyl acetate, methanol and water. The extraction procedure was carried out until the solvent becomes colorless in the soxhlet loop. The extracts were dried using rotary vaccum evaporator and the residue was expressed in terms of dry weight, which was used for further analysis.

About 50 g of leaf powder was soaked in 150 mL of ethanol overnight and filtered. The residue left after filtration was suspended in the same amount of ethanol and left for 48 hours. The two filtrates were mixed, dried using rotatory vacuum evaporator and the residue was stored for further use.

### Qualitative analysis of extracts

Phytochemical analysis of the extracts was performed to detect the presence of constituents such as alkaloids, terpenoids, flavonoids, phenols, saponins, carbohydrates and other metabolites [27,28].

### Thin layer chromatography of extracts

The extracts were examined by thin layer chromatography (TLC) on analytical plates over silica gel (TLC grade, Merck, India). The different solvent systems used for separation were petroleum ether and ethylacetate, hexane and ethylacetate in three different ratios (9:1, 8:2, 7:3); methanol, water, formic acid in the ratio 18:9:1 and chloroform alone. In each case the spots were visualized under ultraviolet light (UV), exposed to iodine vapors and sprayed with vanillin sulfuric acid and ferric chloride solution.

### Enzyme inhibition studies (*In vitro* enzyme inhibition assays)

#### Protease

Protease assay of crude venom was performed according to the method of Greenberg [29]. The reaction mixture composed of 0.5% casein, 1.0 mL of Tris–HCl buffer (pH 8.0), 0.5 mL of 0.25% crude venom and the reaction mixture incubated for four hours at 37°C. At the end of four hours, the reaction was stopped by adding trichloroacetic acid (TCA) and filtered. The filtrate (1.0 mL) was used for protein estimation by the method of Lowry *et al.*[26] using L-tyrosine as a standard. In the above investigation, one unit of enzyme activity was defined as the amount that yielded 0.02 μmole of tyrosine/hour under experimental conditions described. For the inhibition studies, venom was preincubated with the extracts for 30 minutes at 37°C.

#### 5′ nucleotidase

5′ Nucleotidase was assayed by the method of Rowe *et al.*[30]. The substrate solution contained 1 mL of Tris–HCl buffer (pH 8.0), 0.1 mL of 0.1 M magnesium chloride and 0.8 mL of 0.15% 5′AMP followed by 0.25 mL of 0.1% crude venom and incubated at 37°C for 15 minutes. At the end of incubation time, the reaction was quenched by adding TCA and filtered. The filtrate was assayed for inorganic phosphate by the method of Fiske and Subbarow [31] at 625 nm using potassium dihydrogen phosphate as standard. In this analysis, one unit of enzyme activity was defined as the amount that yielded 0.01 μmole of inorganic phosphate/minute under the experimental conditions. For the inhibition studies, venom was preincubated with the extracts for 30 minutes at 37°C.

#### Phosphomonoesterase

The phosphomonoesterase activity was determined by the method of Bessey *et al.*[32] with slight modifications. The reaction mixture included 1.0 mL of Tris–HCl buffer (pH 8.0), 1.0 mL of disodium-p**-**nitrophenol phosphate, 0.5 mL of 0.25% crude venom and was incubated at 37°C for three hours. The absorbance was measured at 425 nm. p-Nitrophenol was used as the standard. One unit of enzyme activity was defined as the amount that yielded 0.1 μmole of p-nitrophenol/hour under the experimental conditions. For inhibition studies, venom was preincubated with the extracts for 30 minutes at 37°C.

#### Phosphodiesterase

Phosphodiesterase activity was determined by a method modified from Lo *et al*. [33]. The assay mixture contained 0.1 mL of venom solution, 0.5 mL of 0.0025 M Na-p-nitrophenyl phosphate, 0.3 mL of 0.01 M MgSO_4_ and 0.5 mL of 0.17 M Tris–HCl (pH 8.0). The absorbance was measured at 400 nm. Phosphodiesterase activity was expressed in nanomoles of product released/minute. Molar extinction coefficient at 400 nm was 8100 Cm^−1^ M^−1^[34]. For the inhibition studies, the venom was preincubated with the extracts for 30 minutes at 37°C.

#### L-amino acid oxidase

The L-amino acid oxidase activity was carried out according to Li *et al*. [35]. Reaction mixture consisted of 1.0 mL of L-leucine, 2.0 mL of Tris–HCl buffer (pH 8.0), 0.25 mL of 0.1% dianisidine hydrochloride, 0.15 mL of 0.1% horseradish peroxidase and 0.04 mL of 0.5% crude venom solution. It was allowed to stand for ten minutes at room temperature and then the absorbance was measured at 415 nm. One unit (U) was defined as the amount of enzyme that catalyzed the formation of 1 μmol H_2_O_2_ per minute. For the inhibition studies, venom was preincubated with extracts for 30 minutes at 37°C.

#### Acetylcholinesterase

Acetylcholine esterase activity was assayed following Ellman *et al.* method [36]. The reaction mixture comprised 3.0 mL of the phosphate buffer (pH 8.0), 10 μL of DTNB (10 mmole/L) and 20 μL of acetylethiocholine iodide (158.5 mmol/L). A total of 50 μL of 0.1% crude venom and 3 mL of buffer solution was incubated at room temperature for five minutes. Then, 10 μL of DTNB (a strong oxidizing agent) and 20 μL of substrate acetylethiocholine iodide were added in order to reach a final concentration of 1 mmole/L. The increase in absorbance at 412 nm was measured on a double beam spectrophotometer against control mixture prepared at the same time. However, in the latter case, 50 μL of enzyme was replaced with 50 μL of buffer solution. For the inhibition studies, venom was preincubated with extracts for 30 minutes at 37°C.

#### Hyaluronidase

Hyaluronidase assay of crude venom was determined turbidometrically by the method of Pukrittayakamee *et al*. [37]. The assay mixture contained buffer Tris–HCl (pH 8.0), 50 μg of hyaluronic acid (0.5 mg/mL in buffer) and enzymes in a final volume of 1.0 mL. The mixture was incubated for 15 minutes at 37°C and the reaction was quenched by the addition of 2 mL of 2.5% (w/v) cetyltrimethylammonium bromide in 2% NaOH (w/v). The absorbance was read at 400 nm (within ten minutes) against a control solution containing 1 mL of the same buffer and 2 mL of 2.5% (w/v) cetyltrimethylammonium bromide in 2% NaOH (w/v). Turbidity reducing activity was expressed as a percentage of the remaining hyaluronic acid, taking the absorbance of a tube in which no enzyme was added as 100%. One unit was defined as the amount of enzyme that provoked 50% turbidity reduction. Specific activity was defined as turbidity reducing units per milligram of enzyme. For the inhibition studies, venom was preincubated with extracts for 30 minutes at 37°C.

#### Phospholipase A_2_

Phospholipase A_2_ assay was determined according to the acidimetric method of Tan and Tan [38] with slight modification. Briefly, a lecithin suspension was prepared with 1% lecithin, 18 mM calcium chloride, and 8.1 mM sodium deoxycholate in equal proportions. The pH of the suspension was adjusted to 8.0 with 1 M sodium hydroxide, and stirred for ten minutes to ensure homogenous mixing. An amount of 0.1 mL of venom solution/fraction was added to 15 mL of egg yolk suspension to initiate the hydrolysis. The initial decrease in pH was measured. A decrease of 1 pH unit corresponded to 133 μmoles of fatty acid release. Enzyme activity was expressed as μmoles of fatty acid released/minute [34]. For the inhibition studies, venom was preincubated with extracts for 30 minutes at 37°C.

## Results and discussion

The extracts were dried using a vacuum evaporator and weight was expressed in terms of dry weight and tabulated in Table 1. The qualitative phytochemical analysis of plant extracts showed significant presence of the metabolites (Table 2). The analysis showed that alkaloids were present only in methanolic extracts which were devoid of carbohydrates, glycosides and resins. The study by Maruthi *et al*. [39] showed similar results except for the presence of carbohydrates. Another study by Muthuswamy *et al.*[40] reported the presence of alkaloids in ethylacetate extract. Changes in the presence of metabolites in various extracts could be due to the time and season in which leaves were collected.

**Table 1:** Weight of the extracts

**Extract**	**Weight in grams (w/w)**
Petroleum ether	0.6
Hexane	0.07
Chloroform	1.14
Ethylacetate	1.25
Methanol	2.65
Water	3.24
Ethanol	2.30

**Table 2:** Qualitative phytochemical analysis of the extracts

**Tests**	**Petroleum ether**	**Hexane**	**Chloroform**	**Ethylacetate**	**Methanol**	**Water**	**Ethanol**
Alkaloids	–	–	–	–	+	–	–
Terpenoids	+	–	+	–	–	–	–
Flavonoids	–	–	+	+	+	–	+
Phytosterols	+	+	+	+	+	+	–
Proteins	–	–	+	+	+	–	–
Carbohydrates	–	–	–	–	–	–	–
Glycosides	–	–	–	–	–	–	–
Phenols	–	–	+	–	–	+	–
Resins	–	–	–	–	–	–	–
Tannins	–	–	–	–	–	+	–
Saponins	–	–	+	–	+	+	+

(+) indicates present, (−) indicates absent.

**Figure 1:**
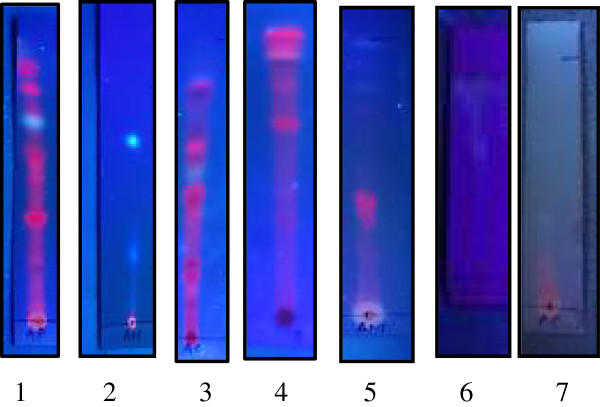
**Thin layer chromatography of the extracts visualized under ultraviolet light.** Lane 1: petroleum ether extract, lane 2: hexane extract, lane 3: chloroform extract, lane 4: ethyl acetate extract, lane 5: methanol extract, lane 6: water extract, and lane 7: ethanol extract.

The thin layer chromatography profiling of all seven extracts provides an insight of the number of phytochemicals present in the extracts. Each extract was separated in a different solvent system according to their polarity and in different ratios of solvent systems. The components in petroleum ether, chloroform and ethylacetate extracts were well separated in petroleum ether and ethylacetate solvent system in the ratio 9:1 and 8:2 respectively. The spots in methanolic extract were well separated in chloroform alone and aqueous extract in methanol, water and formic acid in the ratio 18:9:1. Spots were well defined in ethanolic and hexane extracts with the hexane and ethylacetate solvent system in the ratio 9:1. The visualization of spots was better under UV compared to rest of the agents used. The Figure 1 shows the results under UV light.

**Figure 2:**
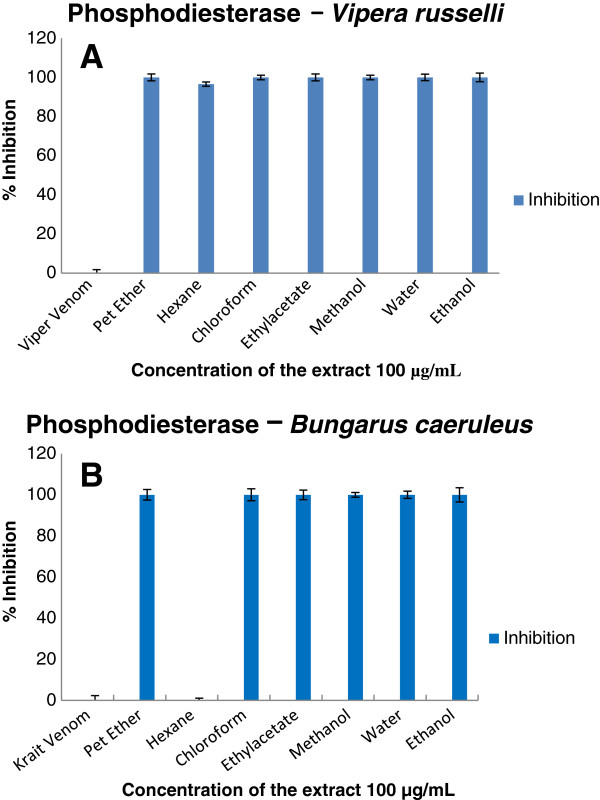
***In vitro *** **inhibition studies of *Azima tetracantha *** **plant extracts against the phosphodiesterase enzyme of (A) *Vipera russelli *** **and (B) *Bungarus caeruleus *** **venom expressed as percent inhibition.**

**Figure 3:**
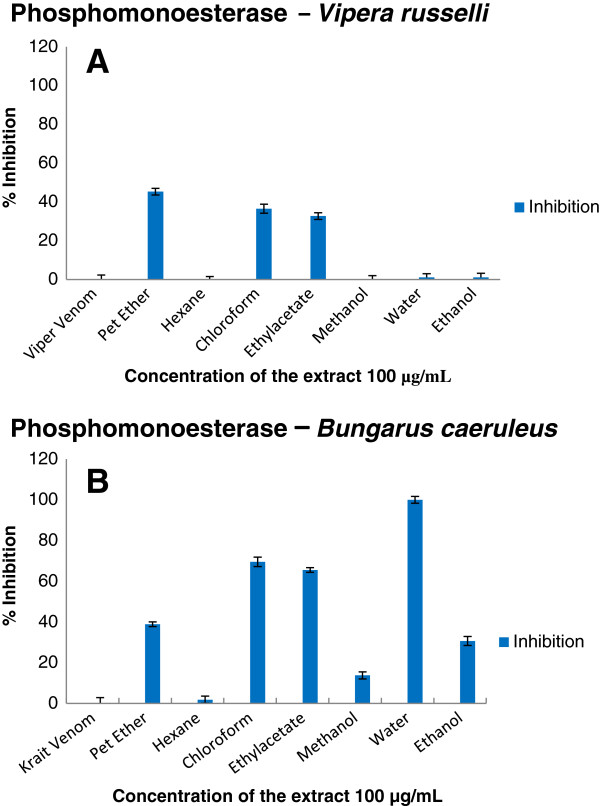
***In vitro *** **inhibition studies of *Azima tetracantha *** **plant extracts against the phosphomonoesterase enzyme of (A) *Vipera russelli *** **and (B) *Bungarus caeruleus *** **venom expressed as percent inhibition.**

**Figure 4:**
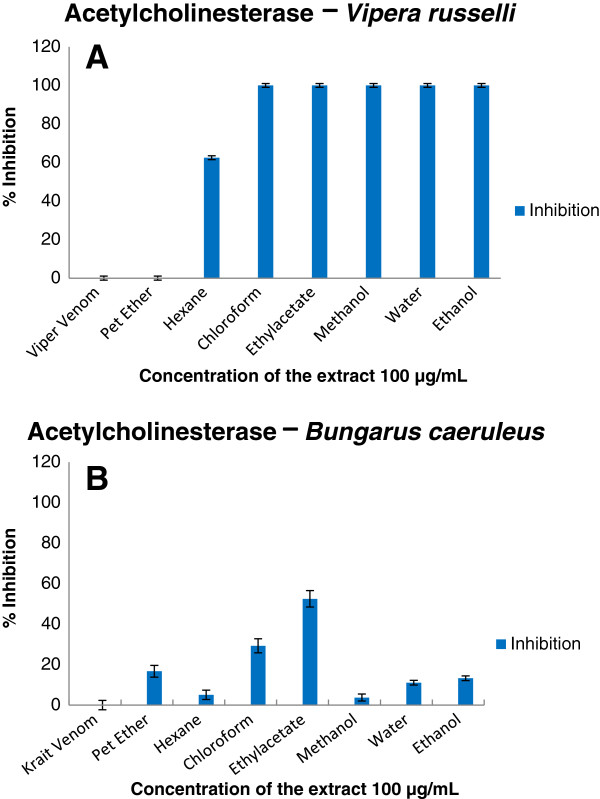
***In vitro *** **inhibition studies of *Azima tetracantha *** **plant extracts against the acetylcholinesterase enzyme of (A) *Vipera russelli *** **and (B) *Bungarus caeruleus *** **venom expressed as percent inhibition.**

**Figure 5:**
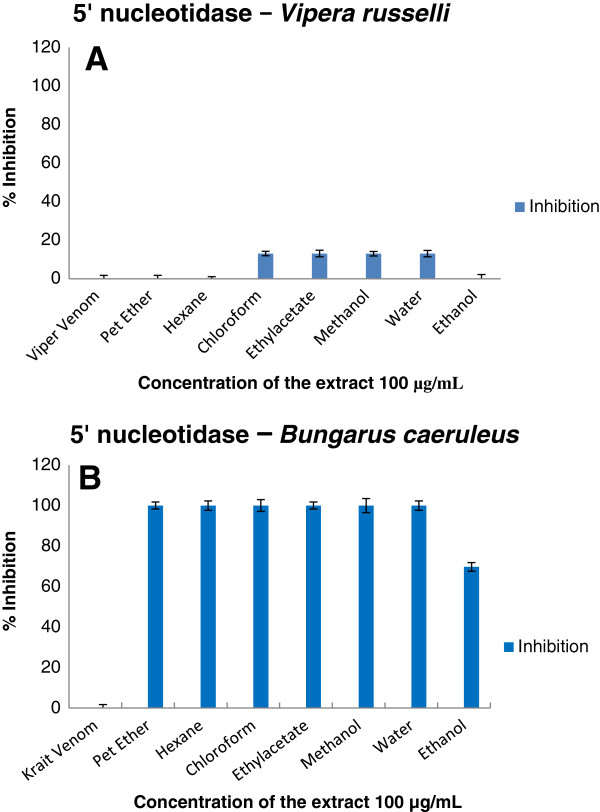
***In vitro *** **inhibition studies of *Azima tetracantha *** **plant extracts against the 5′ nucleotidase enzyme of (A) *Vipera russelli *** **and (B) *Bungarus caeruleus *** **venom expressed as percent inhibition.**

**Figure 6:**
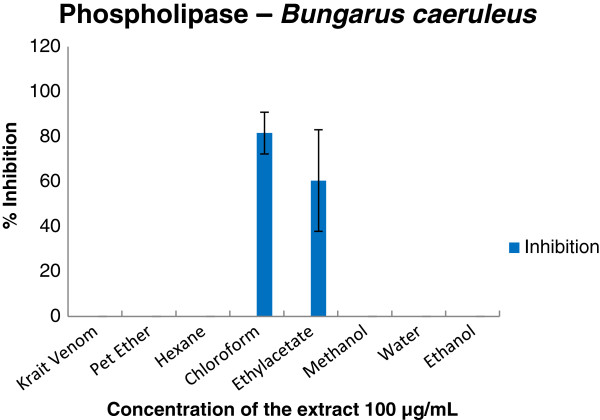
***In vitro *** **inhibition studies of *Azima tetracantha *** **plant extracts against phospholipase A**_**2 **_**enzyme of *Bungarus caeruleus *** **venom expressed as percent inhibition.**

**Figure 7:**
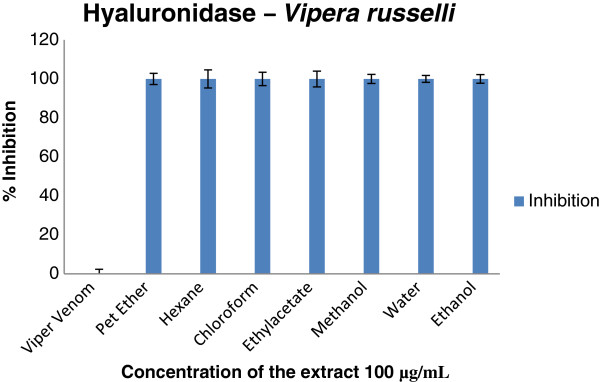
***In vitro *** **inhibition studies of *Azima tetracantha *** **plant extracts against the hyaluronidase enzyme of *Vipera russelli *** **venom expressed as percent inhibition.**

Enzymatic and inhibition studies revealed that the ethylacetate extract of the plant was able to inhibit phosphodiesterase (Figure 2), phosphomonoesterase (Figure 3), acetylcholinesterase (Figure 4), 5′ nucleotidase (Figure 5), phospholipase A_2_ (Figure 6) and hyaluronidase (Figure 7) enzymes present in the venom which renders it as an active extract. The extract is able to inhibit these toxic enzymes in both venoms at 100 μg/mL concentration. The protease and L-amino acid oxidase enzymes were not inhibited by any of the extracts in both venoms. Phospholipase A_2_ and hyaluronidase of *Vipera russelli* and *Bungarus caeruleus* was not inhibited by any of the extracts respectively. The chloroform extract also shows promising results but the acetylcholinesterase was well inhibited in ethyl acetate extract, as it is one of the most toxic enzymes present in venoms of these snakes.

A previous work by Dhananjaya *et al.*[41] has reported the inhibition of enzymes present in Russell’s viper venom by *Mangifera indica* stem bark extract at lower concentrations. The studies by Gopi *et al.*[42] have also reported the inhibition of toxic enzymes present in the *Naja naja* venom by *Andrographis paniculata* active methanolic extract. The present study was performed using a concentration of 100 μg/mL, but the enzyme inhibiting concentration would be as lower as 10 to 500 μL of the diluted extract. Therefore, the actual concentration required to inhibit the enzymes completely should be studied with different concentrations of the active extract.

## Conclusion

In India, snakebite is a major health problem that leads to several deaths annually. *Vipera russelli* and *Bungarus caeruleus* are the most common snakes found throughout the country and a large number of deaths occur due to envenomation by these snakes. Snake antivenom remains the only specific treatment against envenomation by snakes. It is usually derived from horse sera, therefore it contains animal immunoglobulins (that frequently cause complement-mediated side effects), and other proteins (that may cause serum sickness and, occasionally, anaphylactic shock). Although the use of plants against snakebites has been long observed, more scientific attention has been given since to it in the recent 20 years [43]. Several Indian medicinal plants are employed by folk medicine for the treatment of snakebites [6]. In the current study, we examined the antivenom potential of *A. tetracantha* Lam. plant extracts. The inhibition potential of plant extracts in relation to snake toxic enzymes was assayed. The studies were carried out by incubating the venom with plant extracts prior to analysis. The results showed that plant extracts were capable of inhibiting such enzymes. The ethylacetate extract, particularly, showed promising inhibition activity against phosphomonoesterase, phosphodiesterase, acetylcholinesterase, phospholipase A_2_, 5′ nucleotidase and hyaluronidase enzymes, whereas the protease L-amino acid oxidase could not be inhibited. The inhibitory activities of plant extracts against snake venoms should be further confirmed by *in vivo* studies using animal models and by pharmacological analysis *in vitro* including neutralization of fibrinogenolytic activity, neutralization of procoagulant activity and neutralization of hemolytic activity. The authors believe that plant extracts could lead to the development of new drugs against the venom of Indian snakes.

### Ethics committee approval

Venom used in the present study was purchased from a government licensed institute of Tamilnadu state, India, that follows proper procedures regarding animal handling. Doctoral committee of Center for Post Graduate studies and Research, Jain University, Banglore.

## Competing interests

The authors declare that there are no competing interests.

## Authors’ contributions

BJ and MSV worked on the extraction and characterization of phytochemicals in the laboratory. SSM is the corresponding author and designer of the research. KKM helped in the characterization of phytochemicals and enzyme assays by providing suitable suggestions and also helped in characterization of plant species. All authors read and approved the final manuscript.
